# Assessing the responsiveness of musculoskeletal tissues to protein supplementation *in vivo* in older adults: an exploratory randomized controlled trial

**DOI:** 10.1016/j.ajcnut.2026.101349

**Published:** 2026-05-15

**Authors:** Dion CJ Houtvast, Floris K Hendriks, Michelle EG Weijzen, Antoine H Zorenc, Joey SJ Smeets, Pieter J Emans, Will KWH Wodzig, Mads S Larsen, Ulla R Mikkelsen, Martijn GM Schotanus, Bert Boonen, Jeroen MJ van Mulken, Peter AJ de Leeuw, Mireille FM van Stijn, Keith Baar, Luc JC van Loon

**Affiliations:** 1 Department of Human Biology, Institute of Nutrition and Translational Research in Metabolism (NUTRIM), Maastricht University Medical Centre+, Maastricht, The Netherlands; 2 Department of Orthopaedics, Joint Preserving Clinic, Maastricht University Medical Centre+, Maastricht, The Netherlands; 3 Central Diagnostic Laboratory, Maastricht University Medical Centre+, Maastricht, The Netherlands; 4 Arla Foods Ingredients Group P/S, Viby J, Denmark; 5 Department of Orthopaedic Surgery, Zuyderland Medical Center, Sittard-Geleen, The Netherlands; 6 Department of Orthopaedic Surgery, Laurentius Ziekenhuis, Roermond, The Netherlands; 7 Department of Orthopaedic Surgery, Flevoziekenhuis, Almere, The Netherlands; 8 Department of Anaesthesiology, Amsterdam UMC, Amsterdam, The Netherlands; 9 Amsterdam Public Health, Quality of Care, Amsterdam, The Netherlands; 10 Department of Endocrinology and Metabolism, Amsterdam UMC, Amsterdam, The Netherlands; 11 Department of Neurobiology, Physiology and Behavior, University of California Davis, Davis, CA, United States; 12 Department of Physiology and Membrane Biology, University of California Davis, Davis, CA, United States

**Keywords:** musculoskeletal system, protein turnover, heavy water, labeled water, deuterated water, stable isotopes, tracers, collagen

## Abstract

**Background:**

Although muscle protein turnover rates have been well established, little is known regarding the turnover of other musculoskeletal tissues. Furthermore, their response to protein supplementation has not yet been assessed.

**Objectives:**

To assess skeletal muscle, bone, cartilage, tendon, ligament, meniscus, and periarticular soft tissue protein synthesis rates over a 2-wk period with or without additional protein supplementation in vivo in older adults.

**Methods:**

Twenty-four otherwise healthy older adults (12M/12F; age: 68 ± 7 y; body mass index: 29 ± 5 kg/m^2^) undergoing total knee arthroplasty participated in this exploratory randomized controlled trial. Participants were randomly allocated to maintain their habitual diet with (*n* = 12) or without (*n* = 12) an additional daily bolus of 40 g whey protein for 14 d prior to surgery. Deuterium oxide (^2^H_2_O) was administered throughout the 14-d preoperative period, and all musculoskeletal tissues were collected during subsequent surgery to assess tissue protein synthesis rates. Data are presented as means ± standard deviation.

**Results:**

Daily fractional protein synthesis rates (FSRs) in muscle, synovium, and Hoffa’s fat pad averaged 1.17 ± 0.23, 0.79 ± 0.46, and 0.53 ± 0.29%/d, respectively. Fibrous tissues, including anterior and posterior cruciate ligament, patellar tendon, lateral and medial meniscus, and femoral cartilage FSRs averaged 0.45 ± 0.27, 0.46 ± 0.32, 0.20 ± 0.10, 0.21 ± 0.15, 0.18 ± 0.14, and 0.19 ± 0.11%/d, respectively. Finally, FSRs of bone obtained from the distal femur, notch, trochlea, tibia, and patella were 0.18 ± 0.13, 0.21 ± 0.14, 0.13 ± 0.10, 0.12 ± 0.05, and 0.16 ± 0.10%/d, respectively. FSR differed significantly between tissue groups (*P*-tissue group < 0.001), with the highest FSR in muscle, followed by periarticular soft tissues, fibrous tissues, and bone (pairwise comparisons, all *P* < 0.001). Musculoskeletal tissue FSR did not differ between the control and protein supplemented group (*P*-treatment = 0.27, *P*-treatment∗tissue type = 0.14).

**Conclusions:**

Muscle, bone, cartilage, tendon, ligament, meniscus, and periarticular soft tissue protein synthesis rates range between 0.12 and 1.17% per day *in vivo* in older adults. Two weeks of protein supplementation does not seem to increase daily musculoskeletal tissue turnover.

This trial was registered at www.clinicaltrials.gov as NCT03037294.

## Introduction

Skeletal muscle is in a continuous state of turnover, with muscle protein synthesis rates ranging between 1 and 2% per day [1,2]. The dynamic balance between protein synthesis and protein breakdown determines tissue maintenance and allows reconditioning in response to external demands [[2], [3], [4], [5]]. We recently expanded our view by using continuous isotope infusions to assess fasting protein synthesis rates in various other musculoskeletal tissues. In contrast to popular belief, we observed that overnight fasted bone, cartilage, tendon, ligament, and meniscus tissue protein synthesis rates were in the same range as that of skeletal muscle tissue [6]. These findings imply a high level of plasticity in all musculoskeletal tissues.

Physical activity and protein ingestion are potent stimulators of muscle protein synthesis [[7], [8], [9], [10]]. In accordance, a higher protein intake stimulates muscle protein synthesis rates and supports muscle maintenance over the life course [11,12]. Perioperative nutritional support has gained considerable attention over the last decade [[13], [14], [15]]. Current dietary guidelines recommend a daily protein intake of 1.5 g/kg/d in the preoperative and postoperative period to account for illness- and surgery-induced (muscle) protein catabolism [[16], [17], [18], [19]]. Supplementation with protein isolates represents a practical dietary strategy to meet these requirements [17]. However, it remains to be established whether such protein supplementation strategies increase protein synthesis rates of musculoskeletal tissues other than muscle.

The application of deuterium oxide (^2^H_2_O) allows the assessment of tissue protein synthesis rates *in vivo* in humans, under free-living conditions, and over a period of several days to weeks [20,21]. Thus far, this methodology has been applied mainly to study skeletal muscle protein synthesis rates [8,22]. Its application to study the turnover of other musculoskeletal tissues has received little attention, largely due to the complexity of percutaneous sampling of these tissues [23]. However, total knee arthroplasty provides a unique opportunity to sample musculoskeletal tissues that are otherwise not accessible for research. To date, no studies have applied ^2^H_2_O methodology to assess the anabolic responsiveness of a wide range of musculoskeletal tissues to increased protein ingestion.

This study assessed the impact of 14 d of preoperative protein supplementation on muscle, bone, cartilage, tendon, ligament, meniscus, and periarticular soft tissue protein synthesis rates *in vivo* in humans. Twenty-four healthy adults undergoing elective total knee arthroplasty were randomly allocated to consume their habitual diet with or without an additional 40 g whey protein supplement daily for 14 d prior to surgery. Participants ingested ^2^H_2_O throughout this period with tissue samples being collected during subsequent surgery. This study is the first exploratory trial to assess musculoskeletal tissue protein synthesis rates under free-living conditions, with and without additional protein supplementation *in vivo* in humans.

## Methods

### Participants

Twenty-four otherwise healthy adults scheduled to undergo total knee arthroplasty participated in this exploratory randomized controlled trial. Potential participants were identified at the outpatient clinic of the orthopedic departments of participating hospitals within the Netherlands between June 2019 and January 2024. Exclusion criteria were systemic inflammatory diseases, insulin-dependent diabetes mellitus, and the use of medication known to affect protein metabolism (e.g., corticosteroids, biological therapeutics affecting inflammatory pathways and immune function, etc.). Moreover, individuals with secondary osteoarthritis or those who had received an intra-articular corticosteroid injection <3 months prior to surgery were not eligible to participate in the study. All participants were informed about the nature and possible risks of participating in the study before signing written informed consent. The study was approved by the medical ethical committee of the Maastricht University Medical Centre (METC 16-3-036) and boards of all participating hospitals, prospectively registered in clinicaltrials.gov (NCT03037294), and all study procedures were in accordance with the latest version of the declaration of Helsinki.

### Study design

**FIGURE 1:**
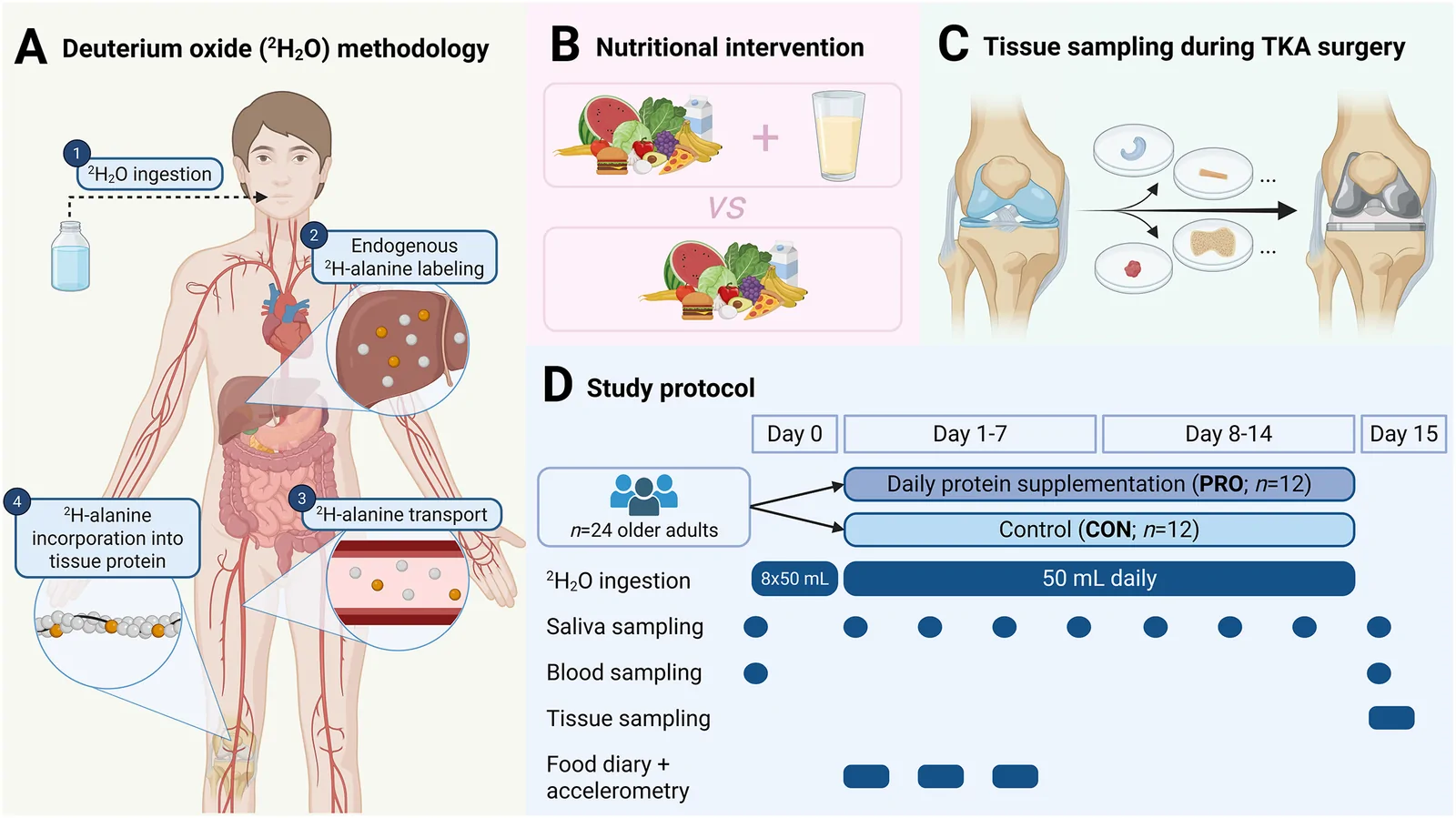
Graphical representation of the experimental design. (A) Deuterium oxide (^2^H_2_O) methodology allows the assessment of tissue protein turnover. For this purpose, participants orally ingest 70% ^2^H_2_O, thereby increasing body water ^2^H enrichments. ^2^H atoms are incorporated into the nonessential amino acid alanine through transamination of pyruvate in the liver. ^2^H-labeled alanine is then transported through the circulation and utilized for the synthesis of new tissue proteins. (B) ^2^H_2_O methodology can be utilized to assess the effects of short-term (dietary) interventions on tissue protein metabolism. (C) Total knee arthroplasty (TKA) allows sampling of numerous musculoskeletal tissues that are resected during surgery. (D) Hence, in this study, 24 older adults undergoing total knee arthroplasty were randomly allocated to maintain their habitual diet with (PRO; *n* = 12) or without (CON; *n* = 12) an additional daily bolus of 40 g whey protein isolate for 14 d prior to surgery. ^2^H_2_O was administered throughout the 14-d preoperative period and all musculoskeletal tissues were collected during subsequent surgery to allow assessment of daily tissue protein synthesis rates. Created with BioRender.com.

A schematic overview of the study design is depicted in Figure 1. Fifteen days prior to surgery (day 0), a medical questionnaire was filled out to assess eligibility. Regarding dietary supplements, none of the participants reported the use of protein supplements, neither currently, nor in the past. Next, baseline blood and saliva samples were obtained and the ^2^H_2_O dosing protocol was initiated. ^2^H_2_O ingestion was continued on days 1 to 14 and participants collected an overnight fasted saliva sample every other day to assess ^2^H enrichments of the body water pool. Participants who were randomly allocated to the protein supplementation group (PRO, *n* = 12) ingested 40 g of whey protein isolate (Lacprodan SP-9225 Instant, Arla Foods Ingredients Group P/S) daily prior to sleep, whereas participants in the control group (CON, *n* = 12) continued their habitual diet. The amino acid composition of the protein supplement is presented in Supplementary Table 1. Participants in the PRO group filled out the time of ingestion on a supplement log and returned the supplement containers to monitor compliance. A block randomization scheme with a block size of 6 was generated using www.randomizer.org by an independent researcher. On the day of surgery (day 15), participants reported to the hospital after which a fasted blood sample was obtained. During the surgical procedure, samples of a large set of different musculoskeletal tissues were collected to assess tissue-specific protein synthesis rates.

### Deuterated water dosing protocol

The ^2^H_2_O dosing protocol consisted of 1 loading day and 14 maintenance days. On day 0, participants ingested 8 boluses of 50 mL 70% ^2^H_2_O (400 mL total; Cambridge Isotopes Laboratories) distributed over the day at 60 to 90-min intervals. On days 1 to 14, participants ingested a single bolus of 50 mL 70% ^2^H_2_O every evening to maintain body water ^2^H enrichments.

### Habitual diet and physical activity

All participants maintained their habitual diet (with the exception of protein supplementation in the PRO group) and physical activity level throughout the study period. No dietary or physical activity restrictions were imposed. Food intake diaries were filled out and an accelerometer (Actigraph GT3X, Actigraph LLC) was worn on 2 weekdays and 1 weekend day to assess habitual dietary intake and physical activity level, respectively. Food intake diaries were filled out on paper and participants were allowed to estimate and describe portion sizes using standard household measures (e.g., cups, tablespoons, etc.). Upon returning the food diary, a member of the study team went over the diary together with the participant to resolve any unclarities. Lastly, the food diaries were analyzed using the web-based software Eetmeter (Voedingscentrum), which contains a built-in food composition database. Mean daily energy and macronutrient intake over the 3-d period were calculated for each participant. In individuals with obesity, relative protein intake was expressed per kg of adjusted rather than actual body weight. Adjusted body weight was calculated as “ideal body weight + (actual body weight − ideal body weight) / 3,” with ideal body weight corresponding to a BMI (in kg/m^2^) of 25 [24]. From the accelerometry data, physical activity parameters were averaged over the 3-d period for each participant.

### Saliva analyses

On day 0, a saliva sample was collected before ingesting the first ^2^H_2_O bolus. On days 1, 3, 5, 7, 9, 11, 13, and 15, saliva samples were collected in the morning following an overnight fast. Participants chewed lightly on a dental cotton swab (Celluron) for ∼1 min until saturated. The cotton swabs were stored in sealed tubes and kept refrigerated until day 15. Saliva was then extracted, snap frozen in liquid nitrogen, and stored at −80°C until further analysis.

Body water ^2^H enrichments were measured as described previously [22]. Briefly, samples were diluted 70-fold and transferred into sealed vials to undergo ^2^H equilibration with hydrogen gas for 24 h at 21°C. The ^2^H enrichment of the hydrogen gas was then measured in duplicate using isotope ratio mass spectrometry (IRMS; DELTA V Advantage IRMS fitted with a GasBench II system and PAL system auto injector; Thermo Fisher Scientific). Standard regression curves were applied from a series of known standard enrichment values against the measured values to assess the linearity of the mass spectrometer and to account for deuterium loss during equilibration.

### Serum analyses

On days 0 and 15, blood samples were collected in serum tubes and allowed to coagulate at room temperature for ≥60 min before centrifugation at 1000 *g* for 15 min. Serum aliquots were snap frozen in liquid nitrogen and stored at −80°C until further analysis. Serum free [^2^H]-alanine enrichments were measured as described previously [22]. Briefly, serum samples were deproteinized and purified after which free amino acids were converted into *tert*-butyldimethylsilyl derivatives before analysis by GC-MS (Agilent 5975C MSD and 7890A GC). Standard regression curves were applied from a series of known standard enrichment values against the measured values to assess the linearity of the mass spectrometer and to account for any isotope fractionation.

### Tissue analyses

Tissue samples of skeletal muscle (*vastus medialis*), synovium, Hoffa’s fat pad, anterior cruciate ligament (ACL), posterior cruciate ligament (PCL), lateral meniscus, medial meniscus, femoral cartilage, distal femoral bone, notch, trochlear bone, tibial plateau bone, patellar bone, and patellar tendon were obtained throughout the surgical procedure. After surgical excision, each tissue sample was collected on a petri dish and carefully processed using a disposable scalpel for soft and fibrous tissues and a bone rongeur for bone. Care was taken to sample only the tissue of interest and ensure a representative, unaffected sample. Samples were then snap frozen in liquid nitrogen and stored at −80°C until further analysis.

Tissue protein–bound [^2^H]-alanine enrichments were measured as described previously [22]. Briefly, 30 to 150 mg wet tissue was freeze dried, weighed, and crushed. Next, ice-cold 2% perchloric acid (PCA) was added and samples were homogenized by sonication. In parallel, 40 μL of 20% PCA was added to 360 μL of serum obtained on day 0 to precipitate the mixed serum protein. This sample was used to measure basal serum protein-bound [^2^H]-alanine enrichments as the background value to assess incremental tissue protein–bound [^2^H]-alanine enrichments [25,26]. All samples were then centrifuged, the supernatants were collected, and the protein pellets were washed 3 times with 1 mL 2% PCA. Samples were hydrolyzed in 3 mL 6 M HCl at 110°C for 15 to 18 h and dried under a nitrogen stream while heated at 110°C. Next, samples were dissolved in 50% acetic acid solution and passed over Dowex exchange resin (AG 50W-X8, 100 to 200 mesh hydrogen form; Bio-Rad) using 2 M NH_4_OH, after which the eluate was dried and the purified amino acids were derivatized to their N(O,S)-ethoxycarbonyl ethyl esters. The derivatized samples were measured using a GC-isotope ratio mass spectrometer (MAT 253; Thermo Fisher Scientific) equipped with a pyrolysis oven using a 60-m DB-17MS column and 5-m precolumn (No. 122– 4762; Agilent) and GC-Isolink. Ion masses 2 and 3 were monitored to determine the ^2^H/^1^H ratios of tissue protein–bound alanine. A series of known standards was applied to assess linearity of the mass spectrometer and to control for the loss of tracer. All analyses were performed in a blinded fashion, ensuring unbiased analysis and data acquisition.

### Calculations

Tissue-specific protein synthesis rates were expressed as fractional synthetic rates (FSR) and calculated using the precursor-product method:

$$\text{FSR}\;\left(\%/d\right)=\left(\frac{E_{p2}-E_{p1}}{E_{\text{precursor}}\times t}\right)\times 100\%$$

where *E*_p1_ and *E*_p2_ are the protein-bound [^2^H]-alanine enrichments in the basal serum (day 0) and in the musculoskeletal tissue samples (day 15), respectively. Furthermore, *E*_precursor_ represents the mean body water ^2^H enrichment corrected by a factor of 3.7 based on the deuterium labeling during *de novo* alanine synthesis [22] and *t* represents the ^2^H_2_O incorporation time.

### Statistical analysis

An *a priori* sample size calculation was performed with muscle protein FSR as a primary outcome measure. On the basis of previous work, we expected a mean difference of 0.008%/h between treatments and an SD of 0.0065%/h in both groups [[27], [28], [29], [30]]. Using a power of 80%, a significance level of 0.05, and a drop-out rate of 10%, the required sample size was calculated as 12 per group. The primary outcome parameter was FSR, which was calculated for each tissue type separately (14 tissues in total). Secondary outcome parameters included dietary energy and macronutrient intake, physical activity measures, body water ^2^H enrichments, serum free [^2^H]-alanine enrichments, and tissue protein–bound [^2^H]-alanine enrichments. Normality and homoscedasticity of the data were confirmed using Shapiro–Wilk tests and visual inspection of the Q–Q plots and histograms.

Participant characteristics, dietary energy and macronutrient intake, and physical activity measures were displayed as descriptive statistics. Two-way repeated measures analysis of variance with treatment as a between-subjects factor was used to assess changes in body water ^2^H enrichments over time. Linear mixed model analysis with treatment and tissue type as fixed factors and subject as a random factor was used to assess the overall effect of protein supplementation on musculoskeletal tissue [^2^H]-alanine enrichments and FSR. Additionally, to assess the effect of protein supplementation on enrichments and FSR of each individual tissue, independent samples *t*-tests were performed. Given the exploratory nature of the study, adjustment for multiple testing was not performed. Finally, tissues were categorized as periarticular soft tissue, fibrous tissue, or bone, and linear mixed model analysis with FSR as the dependent variable, treatment and tissue group as fixed factors, and subject as a random factor was performed. BMI, step count, and dietary protein intake were included as covariates in all linear mixed model analyses. Missing data were not imputed, because linear mixed models use maximum likelihood estimations to use all the available data under the assumption that data are missing at random [31,32]. All data are expressed as means ± SD. Statistical significance was set at *P* < 0.05. All analyses were performed using IBM SPSS Statistics (version 27.0; IBM Corp).

## Results

### Participants

A CONSORT flow diagram is presented in Supplementary Figure 1 [33]. In total, 27 participants enrolled in the study. However, 3 participants in the PRO group had to discontinue participation because their surgery was postponed after initiating the study protocol. Drop-outs were replaced so that 12 participants per group completed the entire protocol. Participant characteristics are presented in Table 1. Compliance with the study protocol was excellent, with 100% of the protein supplements ingested. One participant in the PRO group did not ingest all ^2^H_2_O boluses on the loading day. However, the data were still included in the final analysis, because frequent saliva sampling allowed us to account for the lower precursor enrichments in the FSR calculations.

**TABLE 1:** Participant characteristics

	CON (*n* = 12)	PRO (*n* = 12)
Sex (*n*, female/male)	6/6	6/6
Age (y)	67 ± 6	68 ± 8
Body weight (kg)	90 ± 14	88 ± 21
Height (m)	1.72 ± 0.08	1.76 ± 0.12
BMI (kg/m^2^)	31 ± 6	28 ± 4

Data are expressed as means ± SD.

Abbreviations: CON, control; PRO, daily protein supplementation.

### Physical activity

Physical activity data are presented in Table 2. Daily step counts averaged 6446 ± 3623 steps/d. Moreover, participants spent 73 ± 9% of their waking hours sedentary, and 22 ± 8% and 5 ± 4% engaging in light and moderate-to-vigorous physical activity, respectively.

**TABLE 2:** Habitual physical activity levels

	CON (*n* = 12)	PRO (*n* = 12)
Step count (steps/d)	6657 ± 3063	6236 ± 4238
Sedentary time (min/d)	621 ± 88	670 ± 79
Sedentary time (%)	71 ± 9	76 ± 10
Light activity time (min/d)	215 ± 76	171 ± 60
Light activity time (%)	24 ± 8	19 ± 7
MVPA time (min/d)	45 ± 34	42 ± 41
MVPA time (%)	5 ± 4	5 ± 4

Data are expressed as means ± SD. Participants wore an accelerometer on 2 weekdays and 1 weekend day during the first week of the study period. Data of the 3 d were averaged for each individual participant prior to subsequent analyses.

Abbreviations: CON, control; MVPA, moderate-to-vigorous physical activity; PRO, daily protein supplementation.

### Dietary intake

Self-reported daily energy and macronutrient intake are displayed in Table 3. On average, participants consumed 1737 ± 428 kcal/d. Absolute and relative protein intake averaged 71 ± 17 g/d and 0.9 ± 0.2 g/kg/d, respectively. When including daily protein supplementation, relative protein intake increased to 1.4 ± 0.2 g/kg/d in the PRO group.

**TABLE 3:** Daily energy and macronutrient intake

	CON (*n* = 12)	PRO (*n* = 12)	
		Excl. supplement	Incl. supplement
Energy			
(kcal/d)	1668 ± 374	1805 ± 483	1970 ± 483
Protein			
(g/d)	71 ± 18	72 ± 16	112 ± 16
(g/kg/d)	0.9 ± 0.2	0.9 ± 0.2	1.4 ± 0.2
(En%)	18 ± 4	18 ± 4	25 ± 5
Carbohydrate			
(g/d)	189 ± 56	189 ± 55	189 ± 55
(En%)	47 ± 7	45 ± 6	41 ± 6
Fat			
(g/d)	61 ± 16	71 ± 25	72 ± 25
(En%)	35 ± 5	38 ± 7	34 ± 6

Data are expressed as means ± SD. Participants monitored their dietary intake on 2 weekdays and 1 weekend day during the first week of the study period. Data of the 3 d were averaged for each individual participant before subsequent analyses.

Abbreviations: CON, control; PRO, daily protein supplementation.

### Precursor pool enrichments

**FIGURE 2:**
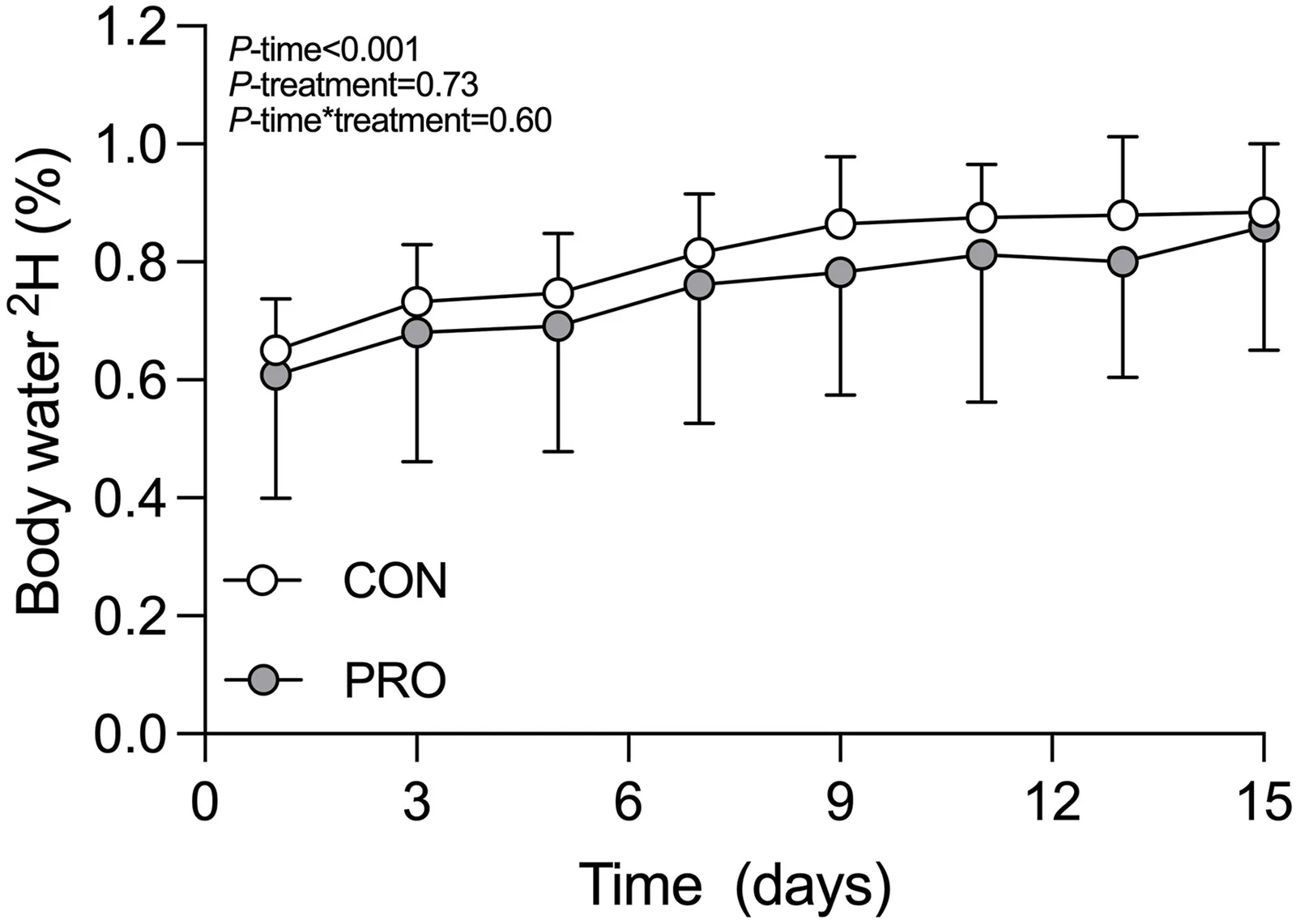
Body water deuterium (^2^H) enrichments over a 2-wk period with daily protein supplementation (PRO) or no-protein control (CON). Participants received 8 × 50 mL 70% deuterium oxide (^2^H_2_O) on day 0, followed by daily 50 mL maintenance doses. Data are presented as means ± SD and were analyzed using 2-way repeated measures ANOVA with treatment as a between-subject factor. ANOVA, analysis of variance.

Body water ^2^H enrichments reached 0.63 ± 0.16% on day 1 and gradually increased to 0.86 ± 0.17% on day 15 (Figure 2; *P*-time < 0.001), with no differences between treatments (*P*-time∗treatment = 0.60). On day 15, serum free [^2^H]-alanine enrichments were 3.10 ± 0.46 mole percent excess (MPE) in CON and 2.76 ± 0.75 MPE in PRO, with no differences between treatments (*P* = 0.19). Moreover, serum free [^2^H]-alanine enrichments were on average 3.4-fold greater than body water ^2^H enrichments on day 15.

### Tissue protein–bound enrichments

On average, protein-bound [^2^H]-alanine enrichments of the soft tissues ranged between 0.22 and 0.49 MPE (Table 4). In contrast, protein-bound [^2^H]-alanine enrichments of the fibrous tissues ranged between 0.07 and 0.20 MPE, whereas bone protein-bound [^2^H]-alanine enrichments ranged between 0.05 and 0.09 MPE. Linear mixed model analysis revealed a significant main effect of tissue type (*P* < 0.001) but no differences between treatments (*P*-treatment = 0.08, *P*-treatment∗tissue type = 0.10). When tested separately, tissue-specific protein-bound enrichments did not differ between PRO and CON, except for medial meniscus enrichments which were higher in CON compared with PRO (*P* = 0.03).

**TABLE 4:** Tissue protein–bound [^2^H]-alanine enrichments in a range of musculoskeletal tissues in and around the knee joint

	CON		PRO	
	*n*	MPE	*n*	MPE
Muscle (*vastus medialis*)	12	0.49 ± 0.12	11	0.48 ± 0.15
Synovium	11	0.40 ± 0.22	12	0.24 ± 0.12
Hoffa’s fat pad	9	0.21 ± 0.12	11	0.22 ± 0.13
Posterior cruciate ligament	6	0.24 ± 0.16	7	0.16 ± 0.14
Anterior cruciate ligament	11	0.16 ± 0.06	12	0.20 ± 0.15
Patellar tendon	10	0.08 ± 0.05	7	0.09 ± 0.05
Lateral meniscus	12	0.10 ± 0.07	12	0.08 ± 0.07
Medial meniscus	12	0.10 ± 0.07	12	0.05 ± 0.03^1^
Femoral cartilage	11	0.08 ± 0.05	12	0.08 ± 0.04
Distal femoral bone	12	0.07 ± 0.05	12	0.08 ± 0.07
Trochlear bone	10	0.06 ± 0.04	12	0.05 ± 0.05
Notch	11	0.09 ± 0.06	10	0.09 ± 0.06
Tibial plateau bone	11	0.05 ± 0.02	12	0.05 ± 0.03
Patellar bone	12	0.08 ± 0.04	8	0.05 ± 0.04

Data are expressed as means ± SD. Availability of tissue samples depends on whether the tissue was resected during surgery or left intact, hence the variable sample sizes. Data were compared between treatments using independent samples *t*-tests. Given the exploratory nature of the study, adjustment for multiple testing was not performed.

Abbreviations: CON, control; MPE, mole percent excess; PRO, daily protein supplementation.

1 Significantly different compared with CON (*P* < 0.05).

### Tissue protein synthesis rates

**FIGURE 3:**
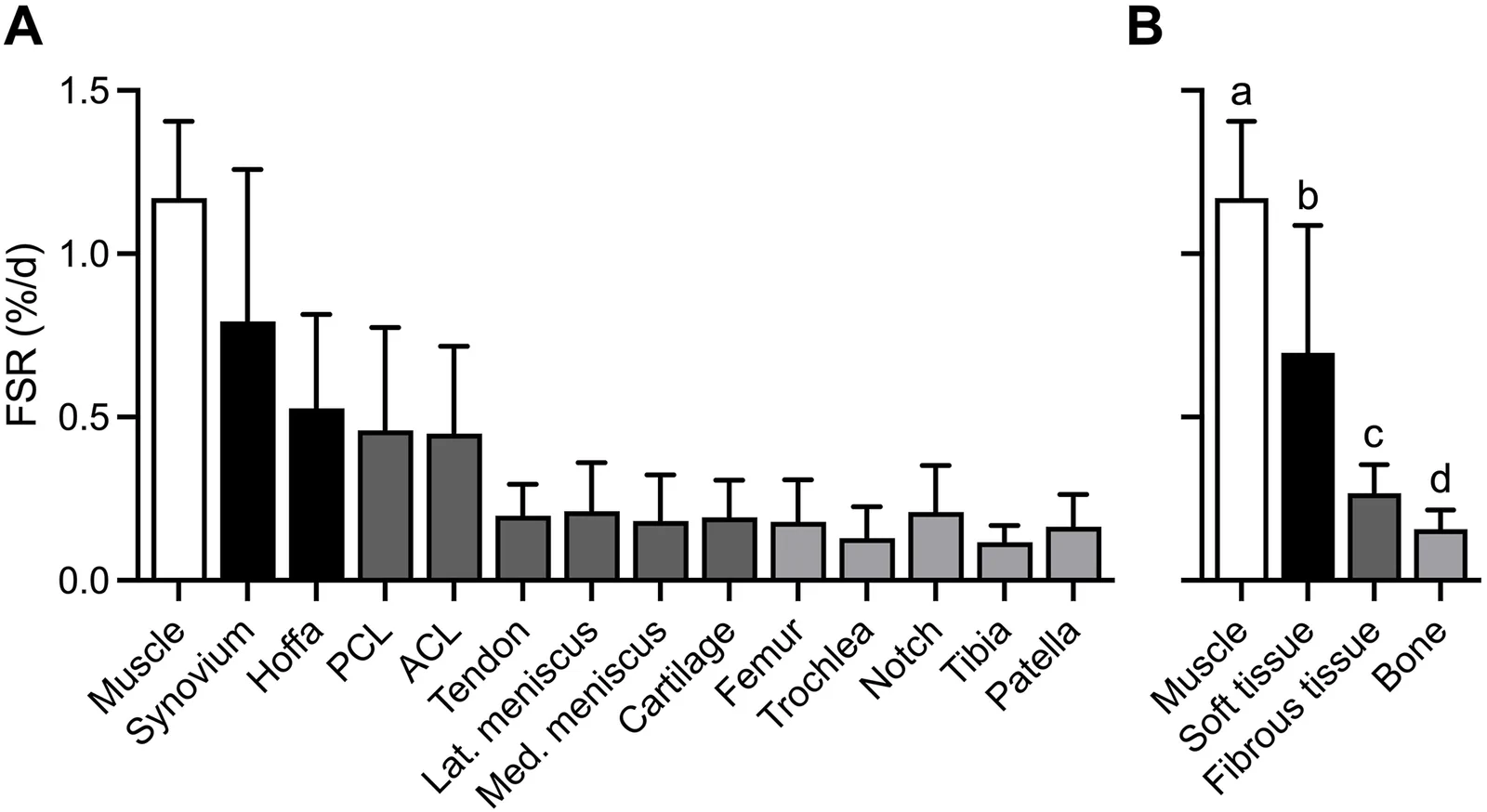
Daily musculoskeletal tissue protein synthesis rates. (A) Fractional synthesis rates (FSR) were based on the incorporation of [^2^H]-alanine into tissue protein and body water ^2^H enrichments were used as a precursor pool. (B) Tissues were grouped based on tissue type, and the average of the tissues within each group was calculated per participant. Data are presented as means ± SD. Tissue groups were compared using linear mixed model analysis. Groups that do not share a common letter differ significantly (pairwise comparisons, all *P* < 0.001). Given the exploratory nature of the study, adjustment for multiple testing was not performed. ACL, anterior cruciate ligament; PCL, posterior cruciate ligament.

**FIGURE 4:**
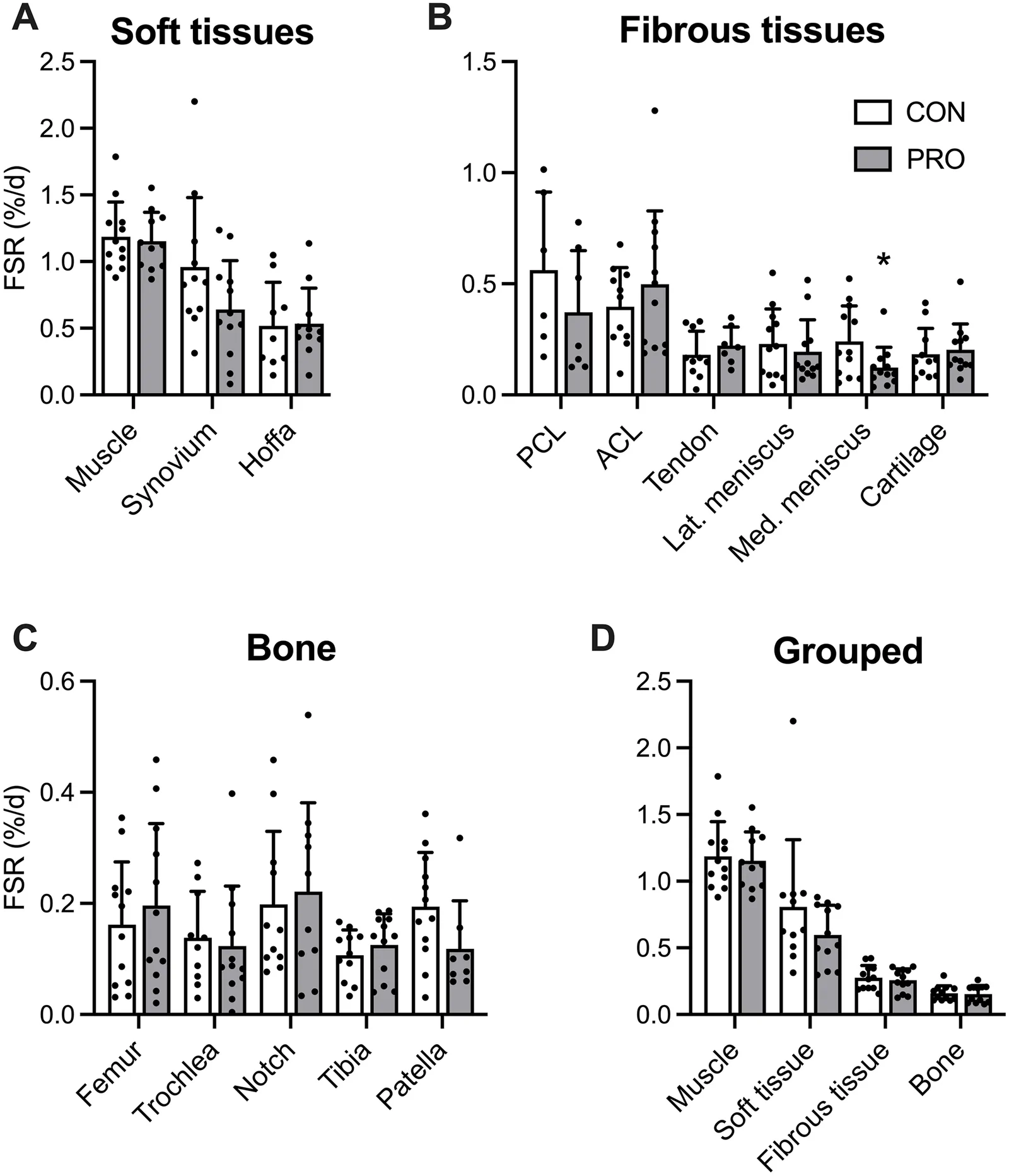
Daily musculoskeletal tissue protein synthesis rates measured over a 2-wk period with daily protein supplementation (PRO) or no-protein control (CON). Fractional tissue protein synthesis rates (FSR) were based on the incorporation of [^2^H]-alanine into tissue protein and body water ^2^H enrichments were applied as precursor pool. Tissues were grouped based on tissue type (panels A–C), and the average FSR of the tissues within each group were calculated per participant (panel D). Data are presented as means ± SD, with dots depicting individual participants. Data were compared using independent samples *t*-tests. Given the exploratory nature of the study, adjustment for multiple testing was not performed. ∗Significantly different compared with CON (*P* < 0.05). ACL, anterior cruciate ligament; PCL, posterior cruciate ligament.

Skeletal muscle (*vastus medialis*), synovium, and Hoffa’s fat pad FSR averaged 1.17 ± 0.23, 0.79 ± 0.46, and 0.53 ± 0.29%/d, respectively (Figure 3A). Fibrous tissues, including ACL, PCL, patellar tendon, lateral and medial meniscus, and femoral cartilage protein synthesis rates averaged 0.45 ± 0.27, 0.46 ± 0.32, 0.20 ± 0.10, 0.21 ± 0.15, 0.18 ± 0.14, and 0.19 ± 0.11%/d, respectively. Finally, protein synthesis rates of bone tissue obtained from the distal femur, notch, trochlea, tibial plateau, and patella averaged 0.18 ± 0.13, 0.21 ± 0.14, 0.13 ± 0.10, 0.12 ± 0.05, and 0.16 ± 0.10%/d, respectively. Linear mixed model analysis showed a significant main effect of tissue type (*P* < 0.001) but no overall differences in musculoskeletal FSR between treatments (Figure 4A–C; *P*-treatment = 0.27, *P*-treatment∗tissue type = 0.14). When tested separately using independent samples *t*-tests, tissue-specific FSR did not differ between treatments, except for medial meniscus FSR, which were higher in CON compared with PRO (*P* = 0.04).

Grouping of the tissues revealed that FSR were significantly different between tissue groups (*P*-tissue group < 0.001), with the highest FSR being observed in muscle (1.17 ± 0.23%/d), followed by periarticular soft tissues (0.70 ± 0.38%/d), fibrous tissues (0.27 ± 0.09%/d), and bone (0.16 ± 0.06%/d; Figure 3B; pairwise comparisons, all *P* < 0.001). Grouped FSR values did not differ between treatments (Figure 4D), neither when tested by linear mixed model (*P*-treatment = 0.17, *P*-treatment∗tissue group = 0.21), nor when tested separately (all *P* > 0.05).

## Discussion

This study provides novel insight into protein turnover in muscle, bone, cartilage, tendon, ligament, meniscus, and periarticular soft tissue under free-living conditions. Using ^2^H_2_O methodology, we show that daily protein synthesis rates are highest in periarticular soft tissues, followed by fibrous tissues and bone, respectively. Furthermore, we demonstrate that 2 wk of daily protein supplementation does not further augment protein synthesis rates in these musculoskeletal tissues.

Basal muscle protein synthesis rates, measured using primed, continuous stable isotope infusion, typically range between 0.04% and 0.06% per hour in an overnight fasted state [1]. Ingestion of a single bolus of dietary protein strongly increases muscle protein synthesis rates, which subsequently return to basal levels after several hours [[34], [35], [36], [37]]. The net result of these postprandial and postabsorptive periods, combined with the impact of physical (in)activity, results in daily muscle protein synthesis rates that range between 1 and 2% per 24 h [8,32,38,39]. In line with this, we present daily muscle protein synthesis rates that averaged 1.2 ± 0.2% per day, as measured over a 2-wk period in otherwise healthy individuals scheduled for total knee arthroplasty.

**FIGURE 5:**
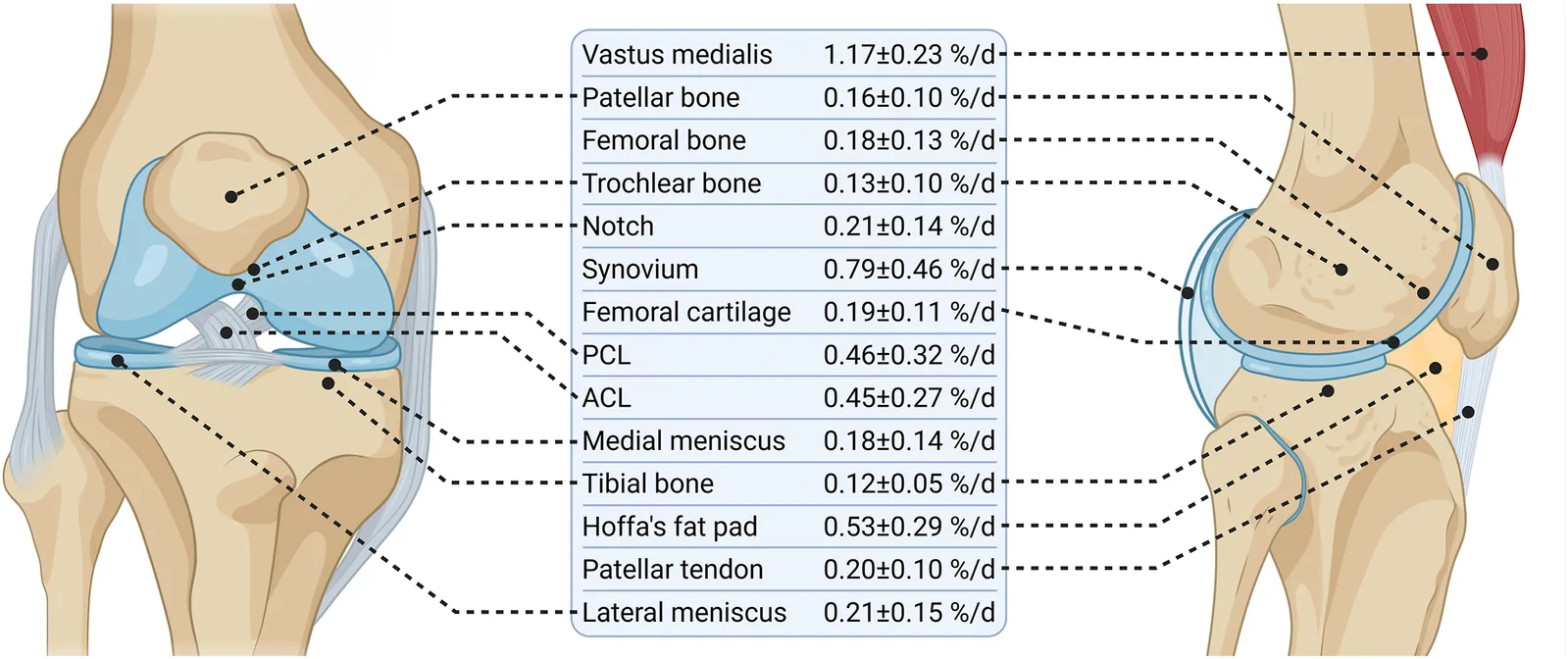
Anatomical depiction of musculoskeletal tissues of the knee joint and their corresponding daily protein synthesis rates (fractional synthesis rates, expressed in %/d). Protein synthesis rates were based on the incorporation of [^2^H]-alanine into tissue protein over a 14-d period with body water ^2^H enrichments as precursor pool. Data are presented as means ± SD. ACL, anterior cruciate ligament; PCL, posterior cruciate ligament. Created with BioRender.com.

Whereas both acute and integrated protein turnover rates are well characterized for skeletal muscle, less is known about the turnover of other musculoskeletal tissues. We extend on our previous work measuring postabsorptive tissue protein synthesis rates [6,40] by establishing daily tissue protein synthesis rates of a wide range of musculoskeletal tissues measured under free-living conditions (Figure 5). Here, we demonstrate that synovium and Hoffa’s fat pad, periarticular soft tissues that play an important role in joint homeostasis, show daily protein synthesis rates ranging between 0.5 and 0.8% per day. Whether these turnover rates can be attributed to structural (i.e., extracellular matrix) remodeling and/or are reflective of metabolic activity remains to be established. To get a better understanding of the relative contribution of such processes to total tissue turnover, future investigations should assess tissue protein synthesis rates of various tissue fractions such as connective proteins and their external milieu [41].

Compared with periarticular soft tissues, fibrous tissues, important for joint stability and force transfer, showed lower daily protein synthesis rates ranging between 0.2 and 0.5% per day. The observed synthetic rates are similar to data previously reported for Achilles tendon (0.24%/d [42]), somewhat higher compared with prior data reported for patellar tendon (0.09%/d [43]), and much higher when compared with a previous study on cartilage turnover (0.03%/d [44]). However, comparability between studies is limited because of different methods used to assess tissue protein synthesis rates under varying conditions as well as over short or more extensive timelines. Although we are well aware of the fluctuations in skeletal muscle tissue protein synthesis rates because of food intake, physical activity, and circadian rhythms, little is known about the impact of such factors on turnover rates of other musculoskeletal tissues. Furthermore, different sample preparation methods may yield different protein subfractions, which may show differences in average synthesis rates because of the proposed existence of fast (immature) and slow (mature) turnover protein pools [45,46]. Although distinguishing between different sets of proteins was beyond the scope of this study, dynamic proteomics approaches could provide further insight into which specific proteins contribute most to bulk protein turnover in these tissues, although at present this remains methodologically challenging [47].

Importantly, absolute tissue protein synthesis rates may differ between previously published studies because of the use of different methods, applied in different settings, and under various conditions. In contrast to previous works, the present data are unique as they provide an extensive overview of the turnover rates of the various musculoskeletal tissues assessed in the same subjects, under the same free-living conditions, using exactly the same methodology. These data allow direct comparisons of turnover rates between the various musculoskeletal tissues under free living conditions *in vivo* in humans (Figure 3).

Similar to fibrous tissues, bone tissue is also largely composed of collagen protein which plays an important role in determining its mechanical properties [48]. In this study, bone displayed the lowest tissue protein synthesis rates, namely 0.1 to 0.2% per day. These rates tend to be in line with those observed in femoral bone in rats [49,50]. To our knowledge, no studies to date have applied ^2^H_2_O methodology to assess bone tissue protein turnover under free-living conditions in vivo in humans, likely due to the difficulty in obtaining bone tissue in humans. Although total knee arthroplasty surgery allowed us to collect numerous bone samples, it should be acknowledged that the turnover rates in the osteoarthritic knee might differ from those in a healthy knee joint. However, by sampling from 5 different regions (with turnover rates all ranging between 0.12% and 0.21% per day), and by carefully selecting sites that did not show any visual signs of pathology, we feel confident that the data represent daily bone protein synthesis rates in older adults. Whether musculoskeletal tissue turnover rates are similar in other age groups and populations remains to be investigated.

Although quantifying protein turnover provides crucial insight into the tissue’s capacity for remodeling and regeneration, we were also interested to assess whether these musculoskeletal turnover rates are responsive to a large increase in daily dietary protein intake. Hence, we randomly allocated participants to either maintain their habitual diet or to consume an additional 40 g of whey protein isolate daily for a period of two weeks. Supplementation effectively increased daily protein intake from 0.9 to 1.4 g/kg/d in the intervention group, thereby approaching current guidelines for perioperative nutrition from the European Society for Clinical Nutrition and Metabolism (ESPEN) [17]. In contrast, participants in the control group showed a daily protein intake of 0.9 g/kg/d, which meets the current recommended dietary allowance (RDA) of 0.8 g/kg/d but has been deemed suboptimal in older adults [51,52]. The additional protein was provided in the evening prior to sleep to introduce an extra meal moment, a dietary strategy that has been shown to be effective to increase daily protein intake in a clinical setting [53]. Furthermore, our laboratory as well as others have shown that the ingestion of 40 g protein, as opposed to 20 g protein, prior to sleep strongly increases overnight muscle protein synthesis rates [54,55]. Despite the theoretical optimal amount, timing, and quality of the ingested protein to stimulate muscle protein synthesis rates, we were unable to detect any significant differences in integrated, daily muscle protein synthesis rates over the 14-d intervention period. Although behavioral changes because of lack of blinding cannot be completely ruled out, our data do not show any meaningful differences in habitual dietary intake and physical activity between groups. Hence, as participants were already consuming ample protein beyond the current RDA, we can only speculate that a further increase in protein intake does not further augment musculoskeletal tissue protein synthesis rates under these free-living conditions.

To date, studies applying ^2^H_2_O methodology in the context of dietary interventions have solely focused on skeletal muscle tissue. Whereas the acute stimulatory effects of protein ingestion on muscle protein synthesis rates are well established, studies on longer-term, integrated muscle protein synthesis rates show mixed results. Although some studies show that a higher daily protein intake leads to greater integrated muscle protein synthesis rates in healthy older men and women [8,56], others have failed to confirm this [38,39]. These discrepancies may be explained by the intervention duration as well as the absolute and relative differences in protein intake between conditions [57]. Other lifestyle factors, such as physical activity, may modulate the proposed impact of the level of protein ingestion on tissue protein turnover. Given that physical activity sensitizes the muscle to the anabolic properties of protein ingestion [58,59], it could be speculated that the level of physical activity will need to be increased before any impact of an increase in protein intake will become evident.

With this study being the first exploratory trial to assess daily musculoskeletal tissue protein synthesis rates with and without additional protein supplementation, more work will be required to confirm the current findings as well as to assess the impact of other dietary, exercise, and/or pharmaceutical interventions on daily protein synthesis rates of various musculoskeletal tissues. For instance, it remains to be established whether hydrolyzed collagen, a protein supplement that more closely resembles the amino acid composition of fibrous tissues and bone, is able to augment musculoskeletal protein synthesis rates [60,61]. Furthermore, besides the impact of such interventions on daily tissue turnover rates, more work is also required to assess the acute effects of nutrient administration on the synthesis rates of musculoskeletal tissues other than muscle [62,63], especially in the context of surgery. Although novel preoperative feeding strategies are promising, more mechanistic insight is needed to further optimize nutritional guidelines that will support postoperative recovery [17,19,64].

In conclusion, integrated daily protein synthesis rates range between 0.5-1.2, 0.2%-0.5, and 0.1-0.2%/d in musculoskeletal soft tissues, fibrous tissues, and bone, respectively, *in vivo* in humans. Based on the findings of this exploratory trial, two weeks of daily protein supplementation does not seem to augment daily protein synthesis rates of these musculoskeletal tissues.

## Author contributions

The authors’ responsibilities were as follows – JSJS, MSL, URM, LJCvL: designed research; DCJH, FKH, MEGW, AHZ, JSJS, PJE, MGMS, BB, JMJvM, PAJdL, MFMvS: conducted research; DCJH, FKH, WKWHW, KB, LJCvL: performed (data) analyses and interpretation; DCJH, LJCvL: wrote the manuscript and had primary responsibility for the final content; and all authors: read and approved the final manuscript.

## Data availability

Data described in the article will be made available upon request, pending application and approval.

## Funding

Arla Foods Ingredients partially funded the work presented. The supporting sources were involved in designing the study but had no involvement in data collection and analyses, nor imposed restrictions regarding publication of the work.

## Declaration of Generative AI and AI-Assisted Technologies in the Writing Process

The author(s) declare that no generative AI or AI-assisted technologies were used in the writing of this manuscript.

## Conflict of interest

MSL and URM are employees of Arla Foods Ingredients. MFMvS has no competing interests to declare for this study. For other research that MFMvS is part of, funding/sponsoring is received from ESAIC, Dutch Medical Food, and Friesland Campina. Both KB and LJCvL and their laboratories have received research grants, consulting fees, speaking honoraria, or a combination of these for research on the impact of exercise and nutrition on muscle metabolism, which include research funding from nutrition companies PepsiCo (KB), GelTor (KB) and those that produce dairy proteins such as Arla Foods Ingredients (LJCvL) and Friesland Campina (LJCvL). A full overview on research funding to LJCvL is provided at https://www.maastrichtuniversity.nl/l.vanloon. All other authors declare no conflicts of interest.

## References

[1] Smith G.I., Patterson B.W., Mittendorfer B. (2011). Human muscle protein turnover--why is it so variable?. J. Appl. Physiol..

[2] Volpi E., Sheffield-Moore M., Rasmussen B.B., Wolfe R.R. (2001). Basal muscle amino acid kinetics and protein synthesis in healthy young and older men. JAMA.

[3] Rennie M.J., Selby A., Atherton P., Smith K., Kumar V., Glover E.L. (2010). Facts, noise and wishful thinking: muscle protein turnover in aging and human disuse atrophy. Scand. J. Med. Sci. Sports.

[4] Kumar V., Atherton P., Smith K., Rennie M.J. (2009). Human muscle protein synthesis and breakdown during and after exercise. J. Appl. Physiol..

[5] Koopman R., van Loon L.J. (2009). Aging, exercise, and muscle protein metabolism. J. Appl. Physiol..

[6] Smeets J.S.J., Horstman A.M.H., Vles G.F., Emans P.J., Goessens J.P.B., Gijsen A.P. (2019). Protein synthesis rates of muscle, tendon, ligament, cartilage, and bone tissue in vivo in humans. PLOS One.

[7] Groen B.B., Horstman A.M., Hamer H.M., de Haan M., van Kranenburg J., Bierau J. (2015). Post-prandial protein handling: you are what you just ate. PLOS One.

[8] McKendry J., Lowisz C.V., Nanthakumar A., MacDonald M., Lim C., Currier B.S. (2024). The effects of whey, pea, and collagen protein supplementation beyond the recommended dietary allowance on integrated myofibrillar protein synthetic rates in older males: a randomized controlled trial. Am. J. Clin. Nutr..

[9] Phillips S.M., Tipton K.D., Aarsland A., Wolf S.E., Wolfe R.R. (1997). Mixed muscle protein synthesis and breakdown after resistance exercise in humans. Am. J. Physiol..

[10] Burd N.A., Tang J.E., Moore D.R., Phillips S.M. (2009). Exercise training and protein metabolism: influences of contraction, protein intake, and sex-based differences. J. Appl. Physiol..

[11] Houston D.K., Nicklas B.J., Ding J.Z., Harris T.B., Tylavsky F.A., Newman A.B. (2008). Dietary protein intake is associated with lean mass change in older, community-dwelling adults: the Health, Aging, and Body Composition (Health ABC) Study. Am. J. Clin. Nutr..

[12] Carbone J.W., Pasiakos S.M. (2019). Dietary protein and muscle mass: translating science to application and health benefit. Nutrients.

[13] Briguglio M., Gianola S., Aguirre M.F.I., Sirtori P., Perazzo P., Pennestri F. (2019). Nutritional support for enhanced recovery programs in orthopedics: future perspectives for implementing clinical practice. Nutr. Clin. Metab..

[14] Williams D.G.A., Wischmeyer P.E. (2020). Perioperative nutrition care of orthopedic surgery patient. Tech. Orthop..

[15] Tedesco A., Sharma A.K., Acharya N., Rublev G., Hashmi S., Wu H.H. (2024). The role of perioperative nutritional status and supplementation in orthopaedic surgery: a review of postoperative outcomes. JBJS Rev.

[16] Volkert D., Beck A.M., Cederholm T., Cruz-Jentoft A., Hooper L., Kiesswetter E. (2022). ESPEN practical guideline: clinical nutrition and hydration in geriatrics. Clin. Nutr..

[17] Weimann A., Braga M., Carli F., Higashiguchi T., Hubner M., Klek S. (2017). ESPEN guideline: clinical nutrition in surgery. Clin. Nutr..

[18] Desborough J.P. (2000). The stress response to trauma and surgery. Br. J. Anaesth..

[19] Hirsch K.R., Wolfe R.R., Ferrando A.A. (2021). Pre- and post-surgical nutrition for preservation of muscle mass, strength, and functionality following orthopedic surgery. Nutrients.

[20] Miller B.F., Reid J.J., Price J.C., Lin H.L., Atherton P.J., Smith K. (2020). CORP: the use of deuterated water for the measurement of protein synthesis. J. Appl. Physiol..

[21] Holwerda A.M., Atherton P.J., Smith K., Wilkinson D.J., Phillips S.M., van Loon L.J.C. (2024). Assessing muscle protein synthesis rates in vivo in humans: the deuterated water ((2)H(2)O) method. J. Nutr..

[22] Holwerda A.M., Paulussen K.J.M., Overkamp M., Smeets J.S.J., Gijsen A.P., Goessens J.P.B. (2018). Daily resistance-type exercise stimulates muscle protein synthesis in vivo in young men. J. Appl. Physiol..

[23] Bergstrom J. (1975). Percutaneous needle biopsy of skeletal muscle in physiological and clinical research, Scand. J. Clin. Lab. Invest..

[24] Thibault R., Abbasoglu O., Ioannou E., Meija L., Ottens-Oussoren K., Pichard C. (2021). ESPEN guideline on hospital nutrition. Clin. Nutr..

[25] Burd N.A., Pennings B., Groen B.B., Gijsen A.P., Senden J.M., van Loon L.J. (2012). The single biopsy approach is reliable for the measurement of muscle protein synthesis rates in vivo in older men. J. Appl. Physiol..

[26] Holm L., Reitelseder S., Dideriksen K., Nielsen R.H., Bulow J., Kjaer M. (2014). The single-biopsy approach in determining protein synthesis in human slow-turning-over tissue: use of flood-primed, continuous infusion of amino acid tracers. Am. J. Physiol. Endocrinol. Metab..

[27] Koopman R., Crombach N., Gijsen A.P., Walrand S., Fauquant J., Kies A.K. (2009). Ingestion of a protein hydrolysate is accompanied by an accelerated in vivo digestion and absorption rate when compared with its intact protein. Am. J. Clin. Nutr..

[28] Koopman R., Walrand S., Beelen M., Gijsen A.P., Kies A.K., Boirie Y. (2009). Dietary protein digestion and absorption rates and the subsequent postprandial muscle protein synthetic response do not differ between young and elderly men. J. Nutr..

[29] Pennings B., Boirie Y., Senden J.M., Gijsen A.P., Kuipers H., van Loon L.J. (2011). Whey protein stimulates postprandial muscle protein accretion more effectively than do casein and casein hydrolysate in older men. Am. J. Clin. Nutr..

[30] Pennings B., Groen B., de Lange A., Gijsen A.P., Zorenc A.H., Senden J.M. (2012). Amino acid absorption and subsequent muscle protein accretion following graded intakes of whey protein in elderly men. Am. J. Physiol. Endocrinol. Metab..

[31] Twisk J., de Boer M., de Vente W., Heymans M. (2013). Multiple imputation of missing values was not necessary before performing a longitudinal mixed-model analysis. J. Clin. Epidemiol..

[32] Domic J., Pinckaers P.J., Grootswagers P., Siebelink E., Gerdessen J.C., van Loon L.J. (2025). A well-balanced vegan diet does not compromise daily mixed muscle protein synthesis rates when compared with an omnivorous diet in active older adults: a randomized controlled cross-over trial. J. Nutr..

[33] Eldridge S.M., Chan C.L., Campbell M.J., Bond C.M., Hopewell S., Thabane L. (2016). CONSORT 2010 statement: extension to randomised pilot and feasibility trials. BMJ.

[34] Pinckaers P.J.M., Kouw I.W.K., Gorissen S.H.M., Houben L.H.P., Senden J.M., Wodzig W. (2023). The muscle protein synthetic response to the ingestion of a plant-derived protein blend does not differ from an equivalent amount of milk protein in healthy young males. J. Nutr..

[35] Gorissen S.H., Remond D., van Loon L.J. (2015). The muscle protein synthetic response to food ingestion. Meat Sci.

[36] Atherton P.J., Etheridge T., Watt P.W., Wilkinson D., Selby A., Rankin D. (2010). Muscle full effect after oral protein: time-dependent concordance and discordance between human muscle protein synthesis and mTORC1 signaling. Am. J. Clin. Nutr..

[37] Trommelen J., van Lieshout G.A.A., Nyakayiru J., Holwerda A.M., Smeets J.S.J., Hendriks F.K. (2023). The anabolic response to protein ingestion during recovery from exercise has no upper limit in magnitude and duration in vivo in humans, Cell Rep. Med.

[38] Stokes T., Mei Y., Seo F., McKendry J., McGlory C., Phillips S.M. (2022). Dairy and dairy alternative supplementation increase integrated myofibrillar protein synthesis rates, and are further increased when combined with walking in healthy older women. J. Nutr..

[39] Murphy C.H., Saddler N.I., Devries M.C., McGlory C., Baker S.K., Phillips S.M. (2016). Leucine supplementation enhances integrative myofibrillar protein synthesis in free-living older men consuming lower- and higher-protein diets: a parallel-group crossover study. Am. J. Clin. Nutr..

[40] Hendriks F.K., Weijzen M.E.G., Goessens J.P.B., Zorenc A.H.G., Gijsen A.P., Kramer I.F. (2023). Trabecular, but not cortical, bone tissue protein synthesis rates are lower in the femoral head when compared to the proximal femur following an intracapsular hip fracture. Bone.

[41] Holwerda A.M., Aussieker T., Senden J., Bouwman F.G., van Kranenburg J., Overman A. (2026). Assessing the composition of myofibrillar and muscle connective protein fractions within skeletal muscle tissue. Crit. Rev. Food Sci. Nutr..

[42] Cramer A., Hojfeldt G., Schjerling P., Agergaard J., van Hall G., Olsen J. (2023). Achilles tendon tissue turnover before and immediately after an acute rupture. Am. J. Sports Med..

[43] Crossland H., Brook M.S., Quinlan J.I., Franchi M.V., Phillips B.E., Wilkinson D.J. (2023). Metabolic and molecular responses of human patellar tendon to concentric- and eccentric-type exercise in youth and older age. Geroscience.

[44] Jorgensen A.E.M., Agergaard J., Schjerling P., Heinemeier K.M., van Hall G., Kjaer M. (2022). The regional turnover of cartilage collagen matrix in late-stage human knee osteoarthritis. Osteoarthr Cartil.

[45] Holm L., Dideriksen K., Nielsen R.H., Doessing S., Bechshoeft R.L., Hojfeldt G. (2019). An exploration of the methods to determine the protein-specific synthesis and breakdown rates in vivo in humans. Physiol. Rep..

[46] Bechshoft C.L., Schjerling P., Borno A., Holm L. (2017). Existence of life-time stable proteins in mature rats-Dating of proteins' age by repeated short-term exposure to labeled amino acids throughout age. PLOS One.

[47] Lim C., McKendry J., Lees M., Atherton P.J., Burd N.A., Holwerda A.M. (2025). Turning over new ideas in human skeletal muscle proteostasis: what do we know and where to from here?. Exp. Physiol..

[48] Burr D.B. (2002). The contribution of the organic matrix to bone's material properties. Bone.

[49] Civil R., Brook M.S., Elliott-Sale K.J., Santos L., Varley I., Lensu S. (2021). A collagen extraction and deuterium oxide stable isotope tracer method for the quantification of bone collagen synthesis rates in vivo. Physiol. Rep..

[50] Civil R., Brook M.S., Santos L., Varley I., Elliott-Sale K.J., Lensu S. (2024). The effects of endurance trainability phenotype, sex, and interval running training on bone collagen synthesis in adult rats. Bone.

[51] Phillips S.M., Chevalier S., Leidy H.J. (2016). Protein "requirements" beyond the RDA: implications for optimizing health. Appl. Physiol. Nutr. Metab..

[52] Deutz N.E., Bauer J.M., Barazzoni R., Biolo G., Boirie Y., Bosy-Westphal A. (2014). Protein intake and exercise for optimal muscle function with aging: recommendations from the ESPEN Expert Group. Clin. Nutr..

[53] Weijzen M.E.G., Kohlen M., Monsegue A., Houtvast D.C.J., Nyakayiru J., Beijer S. (2024). Access to a pre-sleep protein snack increases daily energy and protein intake in surgical hospitalized patients. Clin. Nutr..

[54] Kouw I.W., Holwerda A.M., Trommelen J., Kramer I.F., Bastiaanse J., Halson S.L. (2017). Protein ingestion before sleep increases overnight muscle protein synthesis rates in healthy older men: a randomized controlled trial. J. Nutr..

[55] Groen B.B., Res P.T., Pennings B., Hertle E., Senden J.M., Saris W.H. (2012). Intragastric protein administration stimulates overnight muscle protein synthesis in elderly men. Am. J. Physiol. Endocrinol. Metab..

[56] Oikawa S.Y., Kamal M.J., Webb E.K., McGlory C., Baker S.K., Phillips S.M. (2020). Whey protein but not collagen peptides stimulate acute and longer-term muscle protein synthesis with and without resistance exercise in healthy older women: a randomized controlled trial. Am. J. Clin. Nutr..

[57] Brook M.S. (2025). Investigating muscle protein synthesis using deuterium oxide: the impact of dietary protein interventions across the lifespan. Exp. Physiol..

[58] Pennings B., Koopman R., Beelen M., Senden J.M., Saris W.H., van Loon L.J. (2011). Exercising before protein intake allows for greater use of dietary protein-derived amino acids for de novo muscle protein synthesis in both young and elderly men. Am. J. Clin. Nutr..

[59] Moore D.R., Tang J.E., Burd N.A., Rerecich T., Tarnopolsky M.A., Phillips S.M. (2009). Differential stimulation of myofibrillar and sarcoplasmic protein synthesis with protein ingestion at rest and after resistance exercise. J. Physiol..

[60] Aussieker T., Kaiser J., Hendriks F.K., Janssen T.A.H., Senden J.M., VAN Kranenburg J.M.X. (2025). The effects of ingesting a single bolus of hydrolyzed collagen versus free amino acids on muscle connective protein synthesis rates. Med. Sci. Sports Exerc..

[61] Holwerda A.M., van Loon L.J.C. (2022). The impact of collagen protein ingestion on musculoskeletal connective tissue remodeling: a narrative review. Nutr. Rev..

[62] Babraj J.A., Smith K., Cuthbertson D.J., Rickhuss P., Dorling J.S., Rennie M.J. (2005). Human bone collagen synthesis is a rapid, nutritionally modulated process. J. Bone Miner. Res..

[63] Babraj J.A., Cuthbertson D.J., Smith K., Langberg H., Miller B., Krogsgaard M.R. (2005). Collagen synthesis in human musculoskeletal tissues and skin. Am. J. Physiol. Endocrinol. Metab..

[64] Wischmeyer P.E., Carli F., Evans D.C., Guilbert S., Kozar R., Pryor A. (2018). American Society for Enhanced Recovery and Perioperative Quality initiative joint consensus statement on nutrition screening and therapy within a surgical enhanced recovery pathway. Anesth. Analg..

