# Supplementary material for: Assessing the responsiveness of musculoskeletal tissues to protein supplementation in vivo in older adults: an exploratory randomized controlled trial
Source: Am J Clin Nutr. 2026 May 15;124(1):101349. doi: 10.1016/j.ajcnut.2026.101349 (PMC13494203; doi:10.1016/j.ajcnut.2026.101349)
Supplement: Supplementary file 1 [file mmc1.docx]

**Assessing the Responsiveness of Musculoskeletal Tissues to Protein Supplementation *in vivo* in Older Adults: an Exploratory Randomized Controlled Trial**

Dion CJ Houtvast^1,#^ & Floris K Hendriks^1,#^, Michelle EG Weijzen^1^, Antoine H Zorenc^1^, Joey SJ Smeets^1^, Pieter J Emans^2^, Will KWH Wodzig^3^, Mads S Larsen^4^, Ulla R Mikkelsen^4^, Martijn GM Schotanus^5^, Bert Boonen^5^, Jeroen MJ van Mulken^6^, Peter AJ de Leeuw^7^, Mireille FM van Stijn^8,9,10^, Keith Baar^1,11,12^, Luc JC van Loon^1,*^

SUPPLEMENTARY MATERIAL

**Supplementary Table 1.** Amino acid composition of the protein supplement.

| Amino acid | g AA/100 g protein (Nx6.38) |
| --- | --- |
| Alanine | 6.1 |
| Arginine | 2.2 |
| Aspartic acid (asparagine) | 11.7 |
| Cysteine (cystine) | 2.4 |
| Glutamic acid (glutamine) | 20.1 |
| Glycine | 1.7 |
| Histidine | 1.7 |
| Isoleucine | 7.5 |
| Leucine | 11.3 |
| Lysine | 10.5 |
| Methionine | 2.5 |
| Phenylalanine | 3.0 |
| Proline | 7.2 |
| Serine | 5.5 |
| Threonine | 8.6 |
| Tryptophan | 1.8 |
| Tyrosine | 2.8 |
| Valine | 6.8 |
| AA, amino acid. | |

**Supplementary Figure 1.** CONSORT flow diagram of participant recruitment. PRO, daily protein supplementation; CON, control.
